# IRF8 and MAFB drive distinct transcriptional machineries in different resident macrophages of the central nervous system

**DOI:** 10.1038/s42003-024-06607-6

**Published:** 2024-07-24

**Authors:** Ayato Yamasaki, Iroha Imanishi, Kaori Tanaka, Yasuyuki Ohkawa, Makoto Tsuda, Takahiro Masuda

**Affiliations:** 1 Division of Molecular Neuroimmunology, Medical Institute of Bioregulation, Kyushu University, Fukuoka, Japan; 2 Department of Molecular and System Pharmacology, Graduate School of Pharmaceutical Science, Kyushu University, Fukuoka, Japan; 3 Division of Transcriptomics, Medical Institute of Bioregulation, Kyushu University, Fukuoka, Japan

**Keywords:** Microglia, Neuroimmunology, Microglial cells

## Abstract

The central nervous system (CNS) includes anatomically distinct macrophage populations including parenchyma microglia and CNS-associated macrophages (CAMs) localized at the interfaces like meninges and perivascular space, which play specialized roles for the maintenance of the CNS homeostasis with the help of precisely controlled gene expressions. However, the transcriptional machinery that determines their cell-type specific states of microglia and CAMs remains poorly understood. Here we show, by myeloid cell-specific deletion of transcription factors, IRF8 and MAFB, that both adult microglia and CAMs utilize IRF8 to maintain their core gene signatures, although the genes altered by IRF8 deletion are different in the two macrophage populations. By contrast, MAFB deficiency robustly affected the gene expression profile of adult microglia, whereas CAMs are almost independent of MAFB. Our data suggest that distinct transcriptional machineries regulate different macrophages in the CNS.

## Abstract (web summary)

The authors show that the transcription factors IRF8 and MAFB differentially alter the gene expression profiles of different resident macrophages, which might contribute to their functional diversity within the central nervous system.

## Introduction

The central nervous system (CNS) hosts several macrophage populations, which include microglia that occupy the parenchyma of the CNS, and CNS-associated macrophages (CAMs) that are found at the border, such as meninges, perivascular space, and choroid plexus^1,2^. They both play pivotal roles in the maintenance of tissue homeostasis and the resolution of tissue damage during brain development or diseased condition^1,3^. It has been recently shown that both microglia and CAMs including perivascular macrophages (pvMΦ) and leptomeningeal macrophages (mMΦ), are derived from prenatal progenitors that arise in the yolk sac (YS)^4–6^, which travel to the CNS, followed by acquiring their cellular properties, allowing to perform cell type-specific roles at each territory^1^. Such stepwise development and specification of macrophage populations in the CNS rely on several key transcription factors^4,7,8^. Among them is interferon regulatory factor-8 (IRF8), which contributes to the differentiation of macrophage progenitors in the YS^7^ and the proper distribution of microglia and CAMs during development^9^. Furthermore, global IRF8 knockout dysregulates gene expression patterns in the CNS, causing impaired neuronal functions through enhanced TNF signaling^10^. On the other hand, MAF bZIP transcription factor B (MAFB) was shown to be upregulated in microglia during development, which grants the ability to express the adult gene program and its role in inflammatory regulation^8^. In addition, the opening of genetic loci containing MAF family binding motifs has potentially been shown to be involved in the phenotypic determination of CAMs^11^. However, how strongly these transcription factors contribute to the characterization of adult microglia and CAMs remains unclear.

In the present study, using mouse models with myeloid cell-specific deletion of transcription factors, we examined the roles of IRF8 and MAFB for phenotypic determination of homeostatic microglia and CAMs and found that both populations share IRF8 to keep their core gene signatures with varied dependency. By contrast, although MAFB deficiency robustly affected the gene expression profile of adult microglia, CAMs are almost independent of MAFB in the adult CNS. Our analyses revealed the distinct transcriptional machinery, mediated by IRF8 and MAFB, which underlies the diversity of macrophages in the CNS.

## Results

### Expressions of IRF8 and MAFB in CNS macrophages during development

**Fig. 1:**
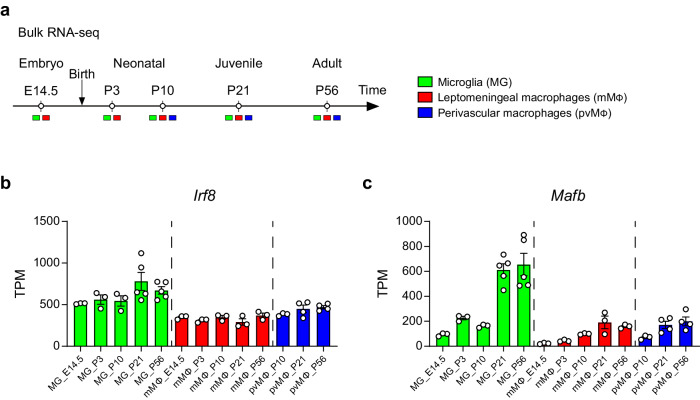
Expressions of *Irf8* and *Mafb* in microglia, perivascular, and leptomeningeal macrophages during development. **a** Scheme of the experimental set-up for bulk RNA-seq at different developmental time points (embryonic day 14.5 (E14.5), postnatal day 3 (P3), P10, P21, and P56). Bar graphs depicting expression levels of *Irf8* (**b**) and *Mafb* (**c**) during development. Data are shown as means of transcripts per million (TPM) ± s.e.m. Each symbol represents one mouse (*n* = 3 for E14.5, P3 and P10 microglia, E14.5, P3, P10, P21 and P56 mMΦ, P10 pvMΦ; *n*  =  4 for P21 and P56 pvMΦ; *n*  =  5 for P21 and P56 microglia).

We first examined the expression pattern of *Irf8* and *Mafb* in microglia and CAMs during development, using our bulk RNA-sequencing (RNA-seq) dataset^9^, for which microglia, pvM, and mMΦ were isolated from *Cx3cr1*^*GFP/+*^ brains at different developmental time-points (Fig. 1a). A stable expression of *Irf8* was observed in all cell types, and the expression level of which was relatively high in microglia, compared to pvMΦ and mMΦ at all timepoints tested (Fig. 1b). In contrast, as previously reported^8^, microglia drastically upregulated the expression of *Mafb* during late development and maintained until adulthood (Fig. 1c), and the expression level of which was higher than that in pvMΦ and mMΦ (Fig. 1c). These data may suggest that the dependency to each transcription factor differs between microglia and CAMs during adulthood.

### Loss of IRF8 or MAFB induces cellular changes in microglia

**Fig. 2:**
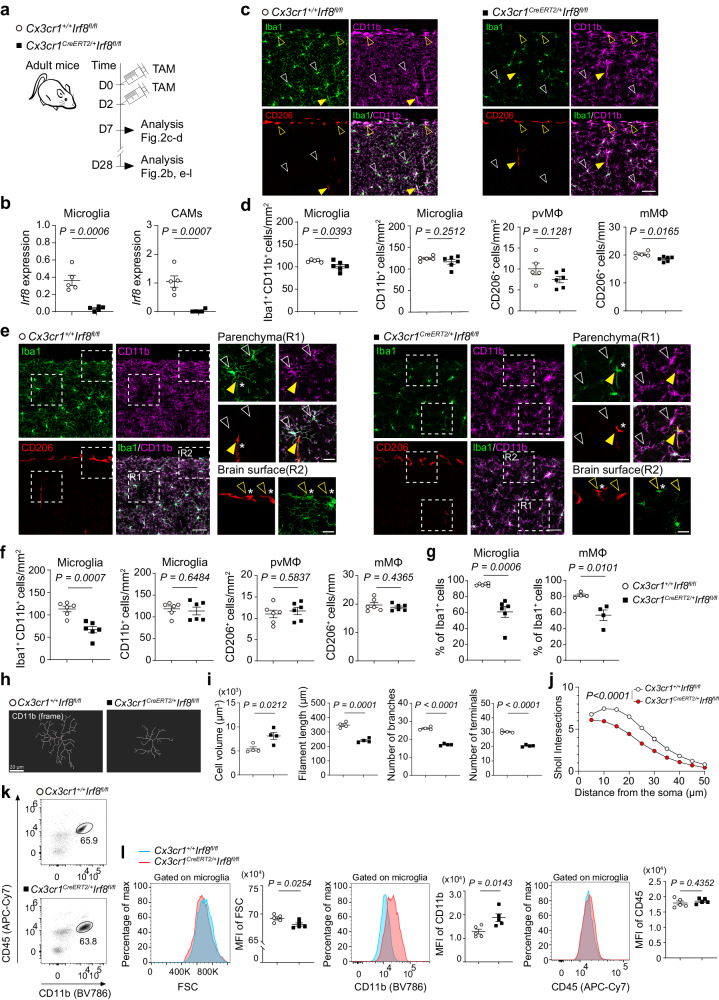
*Irf8* deficiency causes cellular alterations in CNS macrophages. **a** Scheme for the induction of recombination (injection of TAM) and subsequent analysis in *Cx3cr1*^*+/+*^*Irf8*^*fl/fl*^ and *Cx3cr1*^*CreERT2/+*^; *Irf8*^*fl/fl*^ mice. **b** Quantitative PCR of *Irf8* mRNA levels in sorted microglia and CAMs from *Cx3cr1*^*CreERT2/+*^; *Irf8*^*fl/fl*^ and control mice 4 weeks after TAM injection. Each symbol represents one mouse (*n*  =  5). Data are shown as means ± s.e.m. unpaired two-tailed *t*-test. **c** Representative immunofluorescence images from the cortex of *Cx3cr1*^*CreERT2/+*^; *Irf8*^*fl/fl*^ and control *Cx3cr1*^*+/+*^*Irf8*^*fl/fl*^ mice for Iba1 (green), CD11b (magenta) and CD206 (red) depicting microglia (blank white arrowhead), mMΦ (blank yellow arrowhead) and pvMΦ (yellow arrowhead). Scale bars: 50 µm. **d** Quantification of microglia (Iba1^+^CD11b^+^ or CD11b^+^ cells), mMΦ (CD206^+^ cells), and pvMΦ (CD206^+^ cells). Each symbol represents one mouse (*Cx3cr1*^*+/+*^*Irf8*^*fl/fl*^, *n*  =  5; *Cx3cr1*^*CreER2T/+*^*Irf8*^*fl/fl*^, *n*  =  6). Three sections per mouse were quantified. Means ± s.e.m. unpaired two-tailed *t*-test. **e** Representative immunofluorescence images of *Cx3cr1*^*CreERT2/+*^; *Irf8*^*fl/fl*^ and control *Cx3cr1*^*+/+*^*Irf8*^*fl/fl*^ mice for Iba1 (green), CD11b (magenta) and CD206 (red) depicting microglia (blank white arrowhead), mMΦ (blank yellow arrowhead) and pvMΦ (yellow arrowhead). Asterisk indicates Iba1^+^CD206+ cell. Scale bars: 50 µm (main image), 20 µm (inset). **f** Quantification of microglia (Iba1^+^CD11b^+^ or CD11b^+^ cells), mMΦ (CD206^+^ cells), or pvMΦ (CD206^+^ cells). Each symbol represents individual mice (*n*  =  6). Means ± s.e.m. unpaired two-tailed *t*-test. **g** Proportion of Iba1^+^ cells in CD11b^+^ microglia or CD206^+^ mMΦ. Means ± s.e.m. unpaired two-tailed *t*-test. Each symbol represents one mouse (microglia: *n*  =  6, mMΦ: *n* = 4). **h** 3D reconstructed frame images of CD11b^+^ microglia in the cortex. **i** Quantitative assessment of CD11b^+^ microglia. Each symbol represents individual mice (*n*  =  4). 6 regions (30–40 microglia/region) per mouse were quantified. Means ± s.e.m. unpaired two-tailed *t*-test. **j** Sholl analysis plots of microglia (*n*  =  4). Means ± s.e.m. repeated measures two-way ANOVA. **k** Representative fluorescence-activated cell sorting (FACS) plots showing surface expression of CD45 and CD11b. **l** Representative histograms showing forward scatter (FSC), CD11b, and CD45 expression on microglia (left) and mean fluorescence intensity (MFI, right) from *Cx3cr1*^*+/+*^*Irf8*^*fl/fl*^ (blue histogram) and *Cx3cr1*^*CreERT2/+*^; *Irf8*^*fl/fl*^ (red histogram) mice. Each symbol represents one mouse (*n*  =  5). Means ± s.e.m. unpaired two-tailed *t*-test.

**Fig. 3:**
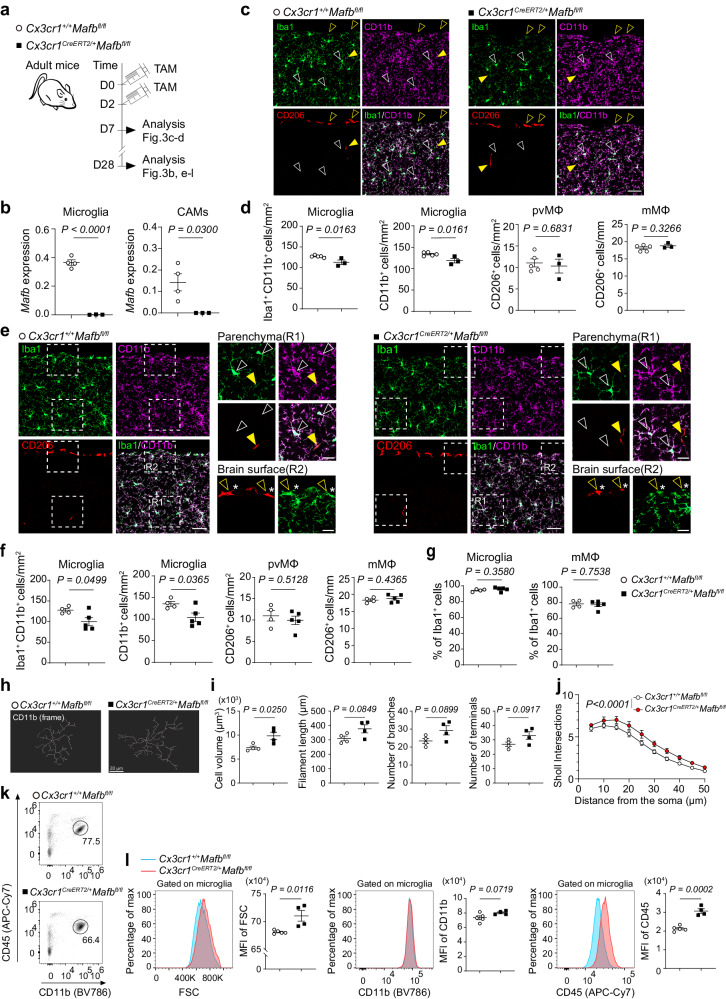
Deletion of *Mafb* results in cellular changes in microglia, but not in CAMs. **a** Scheme for the induction of recombination (injection of TAM) and subsequent analysis in *Cx3cr1*^*+/+*^*Mafb*^*fl/fl*^ and *Cx3cr1*^*CreERT2/+*^*Mafb*^*fl/fl*^ mice. **b** Quantitative PCR of *Mafb* mRNA levels in sorted microglia and CAMs 4 weeks after TAM injection. Each symbol represents one mouse (*n*  =  4 for *Cx3cr1*^*+/+*^*Mafb*^*fl/fl*^, *n*  =  3 for *Cx3cr1*^*CreER2T/+*^*Mafb*^*fl/fl*^). Data are shown as means ± s.e.m. unpaired two-tailed *t*-test. **c** Representative immunofluorescence images from the cortex of *Cx3cr1*^*CreERT2/+*^ *Mafb*^*fl/fl*^ and control *Cx3cr1*^*+/+*^*Mafb*^*fl/fl*^ mice for Iba1 (green), CD11b (magenta) and CD206 (red) depicting microglia depicting microglia (blank white arrowhead), mMΦ (blank yellow arrowhead) and pvMΦ (yellow arrowhead). Scale bars: 50 µm. **d** Quantification of microglia (Iba1^+^CD11b^+^ or CD11b^+^ cells), mMΦ (CD206^+^ cells), and pvMΦ (CD206^+^ cells). Each symbol represents one mouse (*Cx3cr1*^*+/+*^*Mafb*^*fl/fl*^, *n*  =  5; *Cx3cr1*^*CreER2T/+*^*Mafb*^*fl/fl*^, *n*  =  3). Three sections per mouse were quantified. Means ± s.e.m. unpaired two-tailed *t*-test. **e** Representative immunofluorescence images of *Cx3cr1*^*CreERT2/+*^*Mafb*^*fl/fl*^ and control *Cx3cr1*^*+/+*^*Mafb*^*fl/fl*^ mice for Iba1 (green), CD11b (magenta) and CD206 (red) depicting microglia (blank white arrowhead), mMΦ (blank yellow arrowhead) and pvMΦ (yellow arrowhead). Scale bars: 50 µm (main image), 20 µm (inset). Asterisk indicates Iba1^+^CD206^+^ cell. **f** Quantification of microglia (Iba1^+^CD11b^+^ or CD11b^+^ cells), mMΦ (CD206^+^ cells), or pvMΦ (CD206^+^ cells). Each symbol represents individual mice (*Cx3cr1*^*+/+*^*Mafb*^*fl/fl*^, *n*  =  4; *Cx3cr1*^*CreER2T/+*^*Mafb*^*fl/fl*^, *n*  =  5). Means ± s.e.m. unpaired two-tailed *t*-test. **g** Proportion of Iba1^+^ cells in CD11b^+^ microglia or CD206^+^ mMΦ. Means ± s.e.m. unpaired two-tailed *t*-test. Each symbol represents one mouse (*Cx3cr1*^*+/+*^*Mafb*^*fl/fl*^, *n*  =  4; *Cx3cr1*^*CreER2T/+*^*Mafb*^*fl/fl*^, *n*  =  5). **h** 3D reconstructed frame images of CD11b^+^ microglia in the cortex. **i** Quantitative assessment of CD11b^+^ microglia. Each symbol represents individual mice (*n*  =  4). 6 regions (30–40 microglia/region) per mouse were quantified. Means ± s.e.m. unpaired two-tailed *t*-test. **j** Sholl analysis plots of microglia (*n*  =  4). Means ± s.e.m. repeated measures two-way ANOVA. **k** Representative FACS plots showing surface expression of CD45 and CD11b. **l** Representative histograms showing forward scatter (FSC), CD11b, and CD45 expression on microglia (left) and mean fluorescence intensity (MFI, right) from *Cx3cr1*^*+/+*^*Mafb*^*fl/fl*^ (blue histogram) and *Cx3cr1*^*CreERT2/+*^*Mafb*^*fl/fl*^ (red histogram) mice. Each symbol represents one mouse (*n*  =  5 for control, *n*  =  4 for *Cx3cr1*^*CreER2T/+*^*Mafb*^*fl/fl*^). Means ± s.e.m. unpaired two-tailed *t*-test.

To test this and explore cell type-specific roles of IRF8 and MAFB in microglia and CAMs, we crossed mice carrying either a floxed IRF8 (*Irf8*^*fl*^) or MAFB (*Mafb*^*fl*^) with *Cx3cr1*^*CreERT2*^ mice, and adult *Cx3cr1*^*CreERT2*/+^*Irf8*^*fl/fl*^ mice and *Cx3cr1*^*CreERT2*/+^*Mafb*^*fl/fl*^ were administered with tamoxifen (TAM) which allowed for specific deletion of *Irf8* or *Mafb* in myeloid cells including microglia and CAMs (Figs. 2a, b and 3a, b). We then comprehensively examined the cellular features of microglia and CAMs. In comparison with control *Cx3cr1*^+/+^*Irf8*^*fl/fl*^ mice, the number of Iba1^+^CD11b^+^ microglia in *Cx3cr1*^*CreERT2*/+^*Irf8*^*fl/fl*^ mice was markedly decreased 1 and 4 weeks after TAM treatment (Fig. 2c–g). However, the number of microglia single-positive for CD11b was comparable between *Cx3cr1*^*CreERT2*/+^*Irf8*^*fl/fl*^ and *Cx3cr1*^+/+^*Irf8*^*fl/fl*^ littermates (Fig. 2c–f), suggestive of downregulation of Iba1 expression in IRF8-deficient microglia, which is in line with a previous study showing that IRF8 regulates expression of *Aif1* (encoding Iba1) in microglia^12^. Accordingly, the proportion of Iba1^+^ microglia (in CD11b^+^ microglia) were markedly reduced in *Cx3cr1*^*CreERT2*/+^*Irf8*^*fl/fl*^ mice (Fig. 2g). The morphology of microglia was also changed by IRF8 deletion, characterized by increased cell volume and lower cell complexity (Fig. 2h, i), which was further validated by Sholl analysis (Fig. 2j). By contrast, there was no obvious difference in the number and the morphology of CD206^+^ pvMΦ and mMΦ between two genotypes (Fig. 2c–f), with the exception that the proportion of Iba1^+^ mMΦ was markedly decreased in *Cx3cr1*^*CreERT2*/+^*Irf8*^*fl/fl*^ mice (Fig. 2g). We also performed flow cytometric analysis to evaluate the phenotypes of microglia in *Cx3cr1*^*CreERT2*/+^*Irf8*^*fl/fl*^ mice, which exhibited lower forward scatter level and higher CD11b expression (Fig. 2k, l), whereas CD45 expression didn’t differ (Fig. 2l). We next analyzed the brains of *Cx3cr1*^*CreERT2*/+^*Mafb*^*fl/fl*^ mice histologically, and found that *Mafb* deficiency in microglia didn’t affect Iba1 expression, but slightly reduced the cell number which seemed to be more prominent 4 weeks after TAM treatment (Fig. 3c–g), which may suggest an accumulative effect of *Mafb* depletion in microglia. However, no obvious alterations were observed in pvMΦ and mMΦ (Fig. 3c–g). In addition, microglia in the *Cx3cr1*^*CreERT2*/+^*Mafb*^*fl/fl*^ brains showed altered morphology in the opposite direction to those in the brains of *Cx3cr1*^*CreERT2*/+^*Irf8*^*fl/fl*^ mice, with increased cell volume and complexity (Fig. 3h–j). Consistently, flow cytometric analysis revealed a higher value of forward scatter in microglia from *Cx3cr1*^*CreERT2*/+^*Mafb*^*fl/fl*^ mice (Fig. 3k, l). Furthermore, CD45 expression was significantly enhanced after *Mafb* depletion, whereas CD11b expression didn’t change (Fig. 3k, l). Together. these results suggest that IRF8 and MAFB differently contribute to the determination of the phenotypes of microglia and CAMs.

### IRF8 and MAFB differentially contribute to transcriptional regulation in microglia and CAMs

**Fig. 4:**
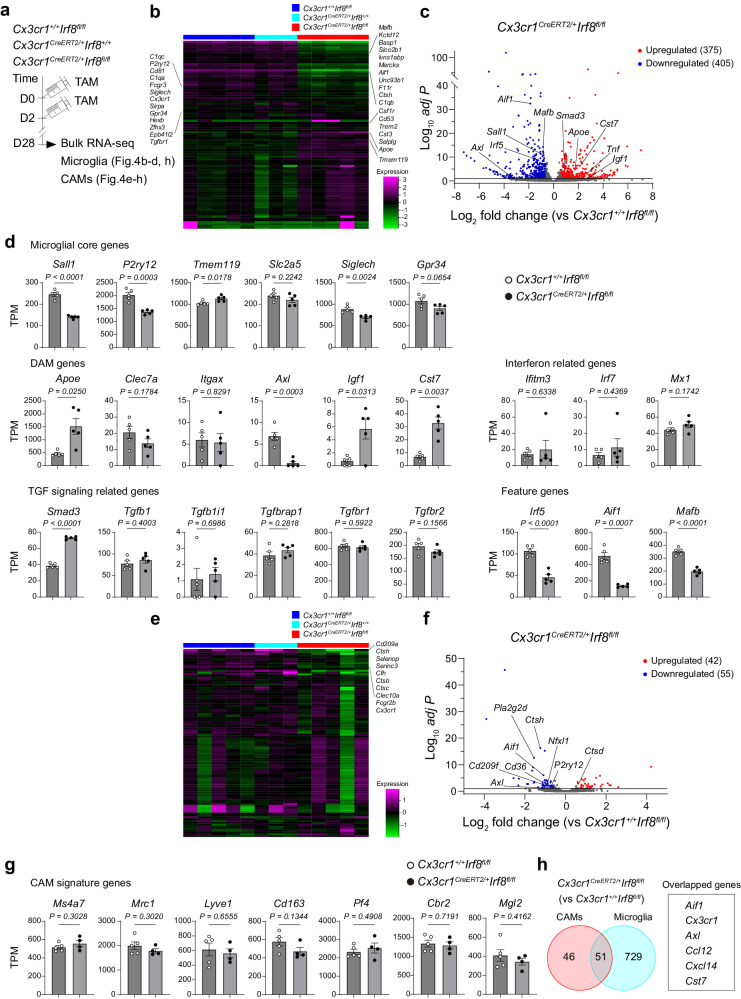
Conditional *Irf8* deletion induces transcriptional changes in CNS macrophages. **a** Scheme for the induction of recombination (injection of TAM) and subsequent analysis in *Cx3cr1*^*+/+*^*Irf8*^*fl/fl*^, *Cx3cr1*^*CreERT2/+*^*Irf8*^*+/+*^ and *Cx3cr1*^*CreERT2/+*^*Irf8*^*fl/fl*^ mice. Heat map of the top 100 differentially expressed genes in microglia (**b**) or CAMs (**e**). olcano plot showing the average log2 fold change and -log10 *adjust P* value for all DEGs between *Cx3cr1*^*+/+*^*Irf8*^*fl/fl*^ and *Cx3cr1*^*CreERT2/+*^*Irf8*^*fl/fl*^ microglia (**c**) or CAMs (**f**). Bar graphs depicting expression levels of selected genes between *Cx3cr1*^*+/+*^*Irf8*^*fl/fl*^ and *Cx3cr1*^*CreERT2/+*^*Irf8*^*fl/fl*^ microglia (**d**) or CAMs (**g**). Means of transcripts per million ± s.e.m. are shown. Each dot represents an individual sample (*n* = 5). unpaired two-tailed *t*-test. **h** Venn diagram representing the number of DEGs shared between CAMs and microglia (*Cx3cr1*^*CreERT2/+*^*Irf8*^*fl/fl*^ vs. *Cx3cr1*^*+/+*^*Irf8*^*fl/fl*^).

**Fig. 5:**
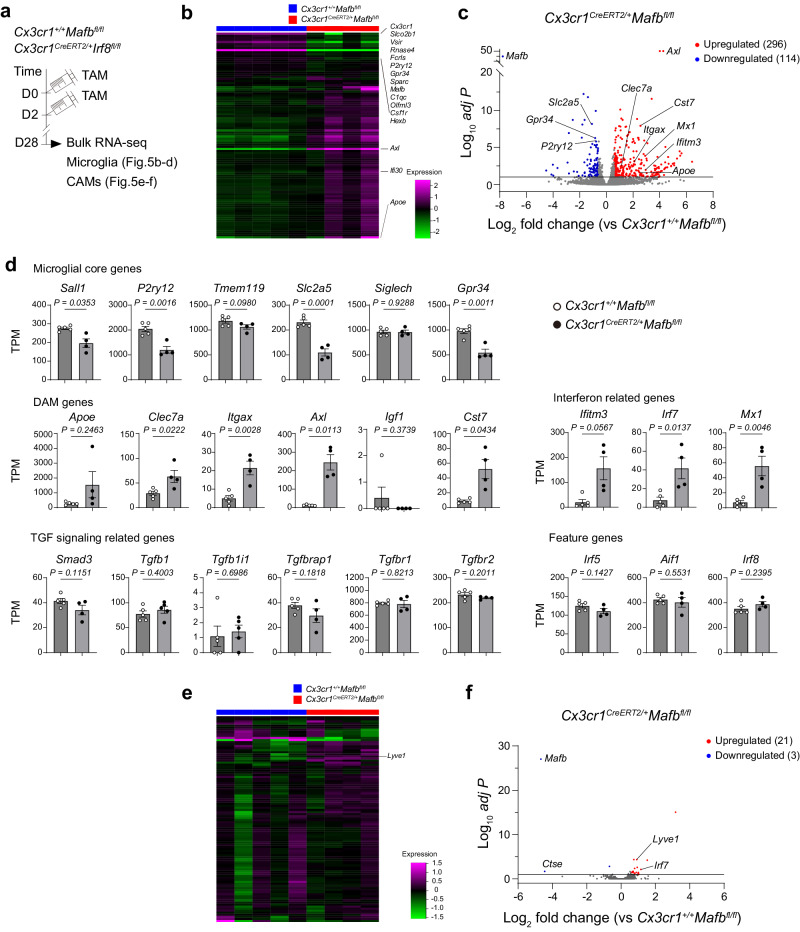
MAFB differently regulates gene expressions in microglia and CAMs. **a** Scheme for the induction of recombination (injection of TAM) and subsequent analysis in *Cx3cr1*^*+/+*^*Mafb*^*fl/fl*^, *Cx3cr1*^*CreERT2/+*^*Mafb*^*+/+*^ and *Cx3cr1*^*CreERT2/+*^*Mafb*^*fl/fl*^ mice. Heat map of the 100 top differentially expressed genes in microglia (**b**) or CAMs (**e**). Volcano plot showing the average log2 fold change and –log10 *adjust P* value for all DEGs between *Cx3cr1*^*+/+*^*Mafb*^*fl/fl*^ and *Cx3cr1*^*CreERT2/+*^*Mafb*^*fl/fl*^ microglia (**c**) or CAMs (**f**). **d** Bar graphs depicting expression levels of selected genes between *Cx3cr1*^*+/+*^*Mafb*^*fl/fl*^ and *Cx3cr1*^*CreERT2/+*^*Mafb*^*fl/fl*^ microglia. Means of transcripts per million ± s.e.m. are shown. Each dot represents an individual sample (*n* = 5 for control, *n* = 4 for *Cx3cr1*^*CreER2T/+*^*Mafb*^*fl/fl*^). unpaired two-tailed *t*-test.

To further investigate the contributions of IRF8 and MAFB to gene regulations, we performed bulk RNA-seq analyses using microglia and CAMs isolated from adult brains of *Cx3cr1*^*CreERT2*/+^*Irf8*^*fl/fl*^ mice or *Cx3cr1*^*CreERT2*/+^*Mafb*^*fl/fl*^ mice 4 weeks after TAM treatment (Figs. 4a and 5a, Supplementary Fig. 1). As compared to *Cx3cr1*^*+*/+^*Irf8*^*fl/fl*^ controls that showed almost comparable gene expression patterns with *Cx3cr1*^*CreERT2*/+^*Irf8*^+/+^ (Fig. 4b), microglia from *Cx3cr1*^*CreERT2*/+^*Irf8*^fl/fl^ mice showed a robust difference in gene expression, with 375 genes being upregulated (Fig. 4c, Supplementary Table 1), consistent with a recent report^13^. As observed in the brains of *Irf8*^-/-^ mice^10^, *Tnf* mRNA was increased. Among the TGF signaling pathway-related genes, Smad3 was significantly upregulated (Fig. 4d). In contrast, 405 genes including microglial core genes including *Sall1* were downregulated (Fig. 4c, d), suggesting an essential role of IRF8 in homeostatic microglia. A marked change in gene expressions in *Cx3cr1*^*CreERT2*/+^*Irf8*^*fl/fl*^ mice was also observed in CAMs, with 51 differentially regulated genes overlapped with those in microglia, such as *Aif1* and *Cx3cr1* (Fig. 4e–h, Supplementary table 1). In addition, *Pla2g2d* (encoding Phospholipase A2 Group IID), *Cd209f* and *Ctsh* (encoding cathepsin h) were downregulated when compared to *Cx3cr1*^*+*/+^*Irf8*^*fl/fl*^ controls (Fig. 4f). To assess the functional insight into the role of IRF8 in microglia and CAMs, we performed gene ontology (GO) enrichment analysis based on differentially regulated genes. As a result, many of the genes altered by IRF8 deletion in microglia and CAMs were immune-related, representing the GO term “Innate immune response” and “Defense response to other organism” (Supplementary Fig. 2). Although the impact of IRF8 depletion on the functions of microglia or CAMs has not been experimentally proven, these data suggest that the functions of homeostatic microglia and CAMs are transcriptionally regulated by IRF8.

On the other hand, analysis of the *Mafb*-deficient microglia revealed that the expression of genes that characterize disease-associated microglia^14,15^, such as *Apoe*, *Itgax*, *Axl*, *Clec7a*, and *Cst7*, or type 1 interferon (IFN)-response genes, such as *Ifitm3*, *Irf7 and Mx1*, were markedly induced when *Mafb* was deleted in microglia (Fig. 5b–d). GO enrichment analysis also showed that genes related to “Defense response” or “Immune response” were upregulated, whereas the GO term enrichment for “Cell motility” or “Locomotion” was downregulated (Supplementary Fig. 3), which may reflect the morphological changes of *Mafb*-null microglia being more ramified (Fig. 3h–j). AXL upregulation in *Mafb*-deficient microglia was confirmed histologically (Supplementary Fig. 4). In addition, downregulation of microglial core genes, such as *Gpr34*, *P2ry12*, and *Slc2a5* (not *Tmem119*), was also evident (Fig. 5b–d). Interestingly, *Irf8*-deficient microglia downregulated the expression of *Mafb* mRNA, whereas *Mafb* deficiency didn’t affect the level of *Irf8* transcripts in microglia (Figs. 4d and 5d), suggesting cell type-specific hierarchical relationship between IRF8 and MAFB in microglia. A previous report has suggested that Maf family transcription factors including MAFB may be involved in the determination of CAM signature^11^. However, unexpectedly, the gene expression profile of CAMs was rarely altered by *Mafb* knockout, with only 24 genes being differentially regulated (Fig. 5e, f and Supplementary Table 1), suggesting the minor contribution of MAFB to the maintenance of homeostatic CAMs. Together, these data suggest that MAFB differently regulates gene expression profiles of microglia and CAMs during homeostatic adulthood.

## Discussion

While the ontogenetical relationship and the gene expression patterns of microglia and CAMs during development have been gradually uncovered, the mechanism by which distinct gene expression patterns between microglia and CAMs are generated, especially the transcriptional regulatory machinery in CAMs, remains poorly understood. In the present study, using mouse models with a conditional gene deletion system, we deeply analyzed the contribution of myeloid lineage-related transcription factors, IRF8 and MAFB, to the maintenance of homeostatic states of microglia and CAMs during adulthood. IRF8 is a crucial factor during brain development for the differentiation of early macrophage progenitors in the yolk sac as well as the proper establishment of CAMs^4,7,9,16^. In addition, microglia in constitutive *Irf8*-deficient mice are known to exhibit several abnormalities in terms of gene expression including Iba1, morphology, and responses to disease-associated events^17,18^. Here, using conditional Irf8 deletion in the adult brains of myeloid cells including microglia and CAMs, we confirmed the importance of IRF8 for the maintenance of homeostatic microglia, as well as CAMs, and the survivability was not affected by Irf8 deletion. Although many genes, such as *Aif1* and *Cx3cr1*, were commonly regulated by IRF8, the dependency of each population on IRF8 differed, which may be due to the different expression levels of IRF8. Nevertheless, a long list of genes was altered in CAMs. Considering that a random effect meta-analysis showed that Irf8 expression was decreased in the brains of schizophrenia^19^ and mutations in IRF8 have been implicated in multiple sclerosis^20^, the changes in Irf8-deficient CAMs might be implicated in the CNS disease progression. Although MAFB is known to control the crucial functions of macrophages^21,22^ and contribute to the pathogenesis of neuropathic pain^23^, its roles in microglia and CAMs remain poorly understood. In this study, we showed that the gene expression profile of homeostatic adult microglia was controlled by MAFB. Unexpectedly, many of the genes altered in *Mafb*-deficient microglia have been categorized as disease-related or type1 interferon-related genes, which are often upregulated in parallel with the changes in microglial state during development, or neurodegenerative diseases or inflammatory conditions^15,24–27^. Although little is known about the role of MAFB in microglia during CNS disease, downregulation of MAFB expression could be a crucial step for driving disease-related gene expression in microglia, which needs further studies to be proven in the future. In contrast, although the opening of genetic loci containing MAF family binding motif has potentially been shown to be associated with the functionality in CAMs^11^, the expression profile of which was barely affected by *Mafb* depletion. Our data strongly suggest the contribution of IRF8 and MAFB to the maintenance of homeostatic microglia and CAMs, but a detailed assessment of the functional changes resulting from the deletion of these factors is lacking and will require further investigation in the future. To date, it remains unknown what transcription machinery determines the state of CAMs in health and disease, especially for the segregation from microglia that are restrictedly controlled with the combination of several transcription factors, such as Sall1 and SMADs^11,28,29^. Thus, uncovering the master regulators for CAMs would be a crucial step to better understand the nature of diverse macrophage populations in the CNS.

## Methods

### Mice

Transgenic lines including *Cx3cr1*^*CreERT2*^ (Jackson Lab, B6.129P2(Cg)-*Cx3cr1*^*tm2.1(cre/ERT2)Litt/*^WganJ, #021160) and *Irf8*^*flox*^ (Jackson Lab, B6.(Cg)-*Irf8*^*tm1.1Hm*^/J, #014175) mice were used in this study. *Mafb*^*flox*^ mice^30^ were kindly provided by Prof. Lisa Goodrich (Department of Neurobiology, Harvard Medical School). Both male and female mice at the age of 8–20 weeks were used. All animal experiments were conducted according to relevant national and international guidelines contained in the ‘Act on Welfare and Management of Animals’ (Ministry of Environment of Japan) and ‘Regulation of Laboratory Animals’ (Kyushu University) and under the protocols approved by the Institutional Animal Care and Use Committee review panels at Kyushu University.

### Tamoxifen treatment

For induction of Cre recombinase activity, tamoxifen (TAM, Sigma-Aldrich, St. Louis, MO) was dissolved in corn oil (Sigma-Aldrich, St. Louis, MO) before injection. In 6- to 10-week-old adult mice, Cre recombination was induced by injecting 4 mg TAM/200 µl oil intraperitoneally twice with a 48-hour interval.

### Flow cytometry

After transcardial perfusion with PBS, brains were homogenized with a potter tissue grinder in Hanks’ balanced salt solution (HBSS) containing 15 mM HEPES buffer and 0.54% glucose, as previously described. The homogenate was separated by gradient centrifugation with 37% Percoll (Sigma-Aldrich, St. Louis, MO) at 800 × *g* for 30 min at 4 °C (no brake). The pellet containing microglia and CAMs at the bottom of the tube was then collected and washed once with PBS containing 2% FBS and 10 mM EDTA before staining. Fc receptors were blocked with Fc block (2.4G2, BD Bioscience) for 10 min at 4 °C before incubation with the primary antibodies. Cells were stained with antibodies directed against CD11b-BV786 (M1/70, BD Bioscience), CD45-APC-Cy7 (30-F11, BioLegend), CD206-APC (C068C2, BioLegend), Ly6C-BV605 (AL-21, BD Bioscience) and Ly6G-PE-Cy7 (1A8, BD Bioscience) for 40 min at 4 °C. After washing, cells were sorted using a CytoFlex SRT (Beckman Coulter). Data were acquired with CytExpert software (Beckman Coulter). Post-acquisition analysis was performed using FlowJo software, version 10.9.0. For quantification of cell surface expression, MFIs were directly compared with unpaired analysis.

### Quantitative PCR (qPCR)

Microglia and CAMs were FACS-sorted from whole brains (see gating strategies used for FACS sorting shown in Supplementary Fig. 1) into a collection tube and then total RNA was purified with the Quick-RNA Micro-Prep kit (ZYMO). For reverse transcription, total RNA was transferred to the reaction with Prime Script reverse transcriptase (Takara, Japan). For quantification of gene deletion, qPCR was performed with TaqMan™ Gene Expression Master Mix (Applied Biosystems) using QuantStudio 3 (Thermo Fisher Scientific). Expression levels were normalized to the values of *Actb*. The sequences of TaqMan primer pairs and probes are described below.

#### MAFB

Forward primer: 5′-ACCTAGACCTCCCCTATA ACTAC-3′

Reverse primer: 5′-ACTAACGCTGCAACTCTCAAG-3′

TaqMan probe: 5′-/56-FAM/ACCATTAAG/ZEN/TCTCCCTG TCTCCAGA/3IABkFQ/-3′

#### IRF8

Forward primer: 5′-TGTCTCCCTCTTTAAACTTCCCG-3′

Reverse primer: 5′-GAAGACCATGTTCCGTATCCC-3′

TaqMan probe: 5′-/56-FAM/ACCTCCTGA/ZEN/TTGTAATCCTGCTTGCC/3IABkFQ/G-3′

#### ACTB

Forward primer: 5′-GACTCATCGTACTCCTGCTTG-3′

Reverse primer: 5′-GATTACTGCTCTGGCTCCTAG-3′

TaqMan probe: 5′-/56-FAM/CTGGCCTCA/ZEN/CTGTCCACCTTCC/3IABkFQ/-3′

### Bulk RNA-seq

RNA-seq of the 3’-untranslated region was performed using the CEL-Seq2 protocol^31^, except that MaximaH minus reverse transcriptase (Thermo Scientific, #EP0751) was used for single-stranded synthesis, and the Second Strand Synthesis Module (NEB, #E6111) was used for double-stranded cDNA synthesis; amplification was performed by nine cycles of PCR without sample pooling. Sequencing was performed on an Illumina NovaSeq 6000, and the subsequent quantitative analysis was performed using 81 bp of insert reads (Read2). Adapter sequences and low-quality sequences were removed, and read lengths less than 20 bp were discarded using Trim Galore (ver. 0.6.10). Then, reads were mapped to the GRCm38 reference using HISAT2 (ver. 2.2.1). Read counts for each gene were obtained using featureCounts (ver. 2.0.4), and DEGs were extracted with DESeq2 (ver. 1.34.1) or iDEP (ver. 0.96) using |FC| > 1.5 and padj < 0.1 as threshold values.

### Immunohistochemistry

After transcardial perfusion with PBS, brains were fixed 7-hour in 4% PFA, dehydrated in 30% sucrose, and embedded in Tissue-Tek O.C.T. compound (Sakura Finetek). As previously described^9^, cryosections were cut at 20 µm thickness and were then blocked with PBS containing 5% bovine serum albumin and permeabilized 0.5% Triton-X 100 (MP Biomedicals) in blocking solution at RT. After that, tissue sections were incubated for 2 days at 4 °C with primary antibody for Iba1 (1:1000 018-28523, FUJIFILM Wako; 1:1000 234 308, Synaptic Systems), CD206 (MCA2235, Biorad), Collagen IV (1:200 AB769, Millipore), CD11b (1:1000 MCA711G, Biorad) and AXL (1:500 AF854, R&D systems). Secondary antibodies were purchased from Thermo Fisher Scientific or Jackson Laboratory and added as follows: Alexa Flour^®^ 405 1:1000, Alexa Flour^®^ 488 1:1000, Cy3 1:1000, and Alexa Fluor^®^ 647 1:1000 for 3 h at RT. Coverslips were mounted ProLong^TM^ Glass Antifade Mountant with/without NucBlue^TM^ (Invitrogen). Images were taken using a LSM900 (Carl Zeiss) or BZ-X810 (Keyence).

### Cell quantifications

To assess the density of cells, the number of Iba1^+^ CD206^−^ (microglia) or CD206^+^ cells (pvMΦ and mMΦ) were quantified on a fluorescence microscope (BZ-X810). Microglia and pvMΦ were normalized to the area of the region of interest and expressed as cells / mm^2^. mMΦ were normalized to the length of the leptomeninges indicated by collagen IV immunofluorescence and finally expressed as cells/mm^2^. At least three sections of a minimum of four mice were used for each analysis. For morphological change analysis, microglia images were captured using a confocal laser scanning microscope (LSM900, Carl Zeiss) and were analyzed using IMARIS software (Version 10.0, Oxford Instrument). To access morphological complexity, Sholl Analysis was performed using filament reconstruction mode in IMARIS. The intensity of microglial AXL was calculated as the summation of AXL fluorescence intensity within CD11b^+^ cells using IMARIS by creating a 3D surface rendering of individual microglia.

### Statistics and reproducibility

Statistical significance was determined using the unpaired two-tailed Student’s *t* test, Welch’s *t* test, or repeated measures two-way analysis of variance (ANOVA) using GraphPad Prism 9.5.1. The number of replicates is defined in each figure legend.

### Supplementary information

Supplementary Information

Description of additional supplementary files

Supplementary Data 1

Supplementary Table 1

reporting-summary

## Data Availability

The bulk RNA-sequencing data related to Fig. 1 are available in the previous paper^9^. The other raw data for mouse bulk RNA-sequencing have been deposited in the Gene Expression Omnibus, and are available at the following accession number: GSE269745. All the numerical source data can be found in the “Supplementary Data 1” file associated with the manuscript. All other data that support the findings of this study are available from the corresponding authors upon reasonable request.
