# Supplementary material for: IRF8 and MAFB drive distinct transcriptional machineries in different resident macrophages of the central nervous system
Source: Commun Biol. 2024 Jul 24;7:896. doi: 10.1038/s42003-024-06607-6 (PMC11266354; doi:10.1038/s42003-024-06607-6)
Supplement: Supplementary file 1 — Supplementary Information [file 42003_2024_6607_MOESM1_ESM.pdf]

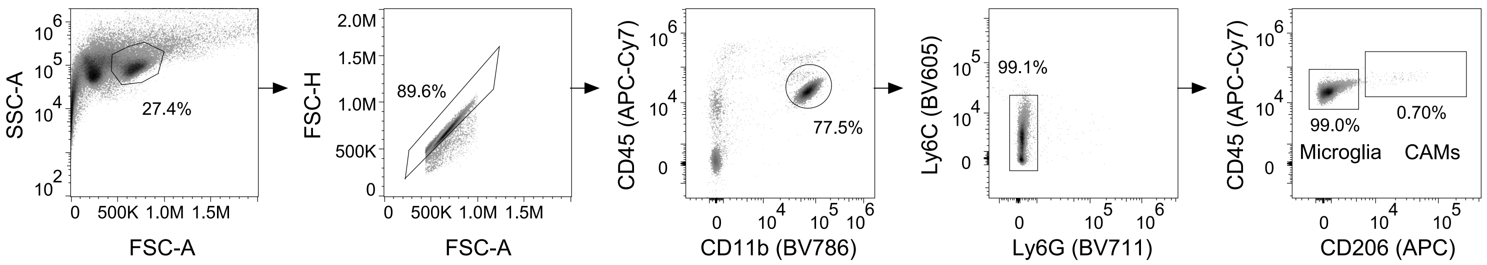

Supplementary Fig. 1: Gating strategies for microglia and CAMs sorting.

Representative gating strategy for FACS-sorting of $CD11b^{+}$ $CD45^{+}$ $Ly6G^{-}$ $CD206^{-}$ microglia and $CD11b^{+}$ $CD45^{+}$ $Ly6G^{-}$ $CD206^{+}$ CAMs from whole brain for bulk RNA-seq.

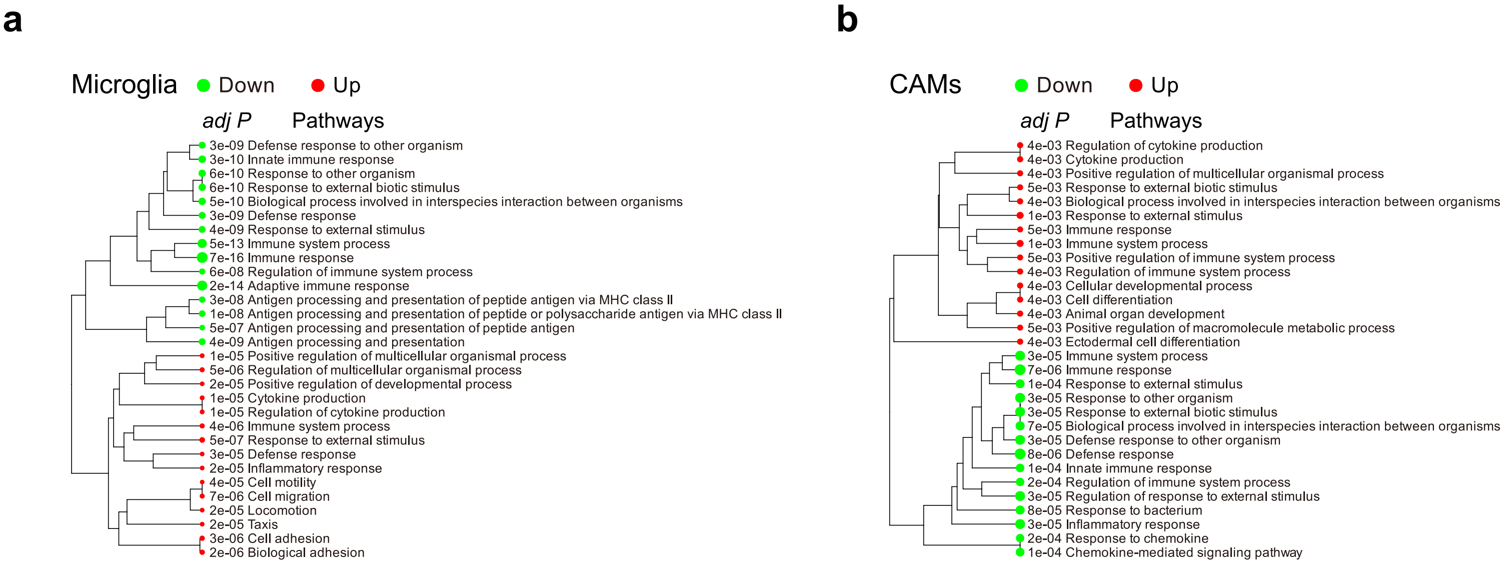

Supplementary Fig. 2: GO enrichment analysis of microglia and CAMs in *Irf8*-deficient mice. Gene Ontology analysis of differentially expressed genes (DEGs) in *Cx3cr1*$^{CreERT2/+}$*Irf8*$^{fl/fl}$ microglia (**a**) or CAMs (**b**). Selected terms are represented in a dot plot, with the size of the dot corresponding to the *adj P* value of enrichment and the color of dots corresponding to the direction of pathways.

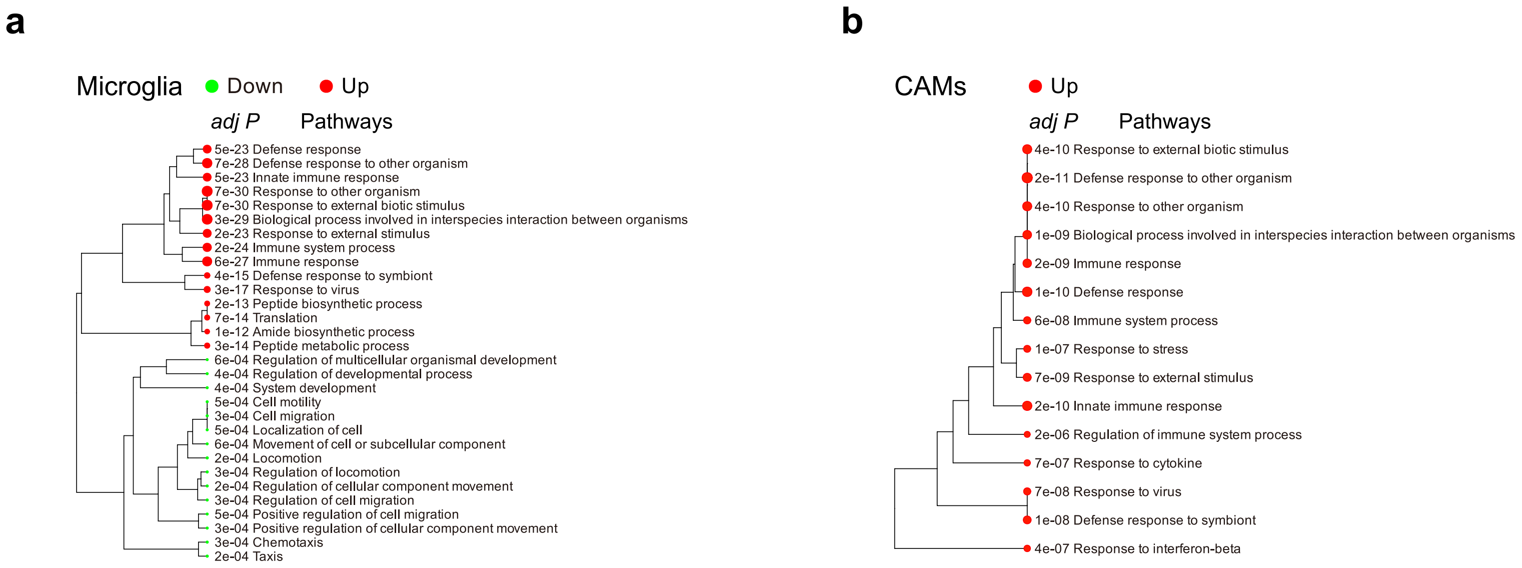

Supplementary Fig. 3: GO enrichment analysis of microglia and CAMs in *Mafb*-deficient mice.

Gene Ontology analysis of differentially expressed genes (DEGs) in *Cx3cr1*$^{CreERT2/+}$*Mafb*$^{fl/fl}$ microglia (**a**) or CAMs (**b**). Selected terms are represented in a dot plot, with the size of the dot corresponding to the *adj P* value of enrichment and the color of dots corresponding to the direction of pathways.

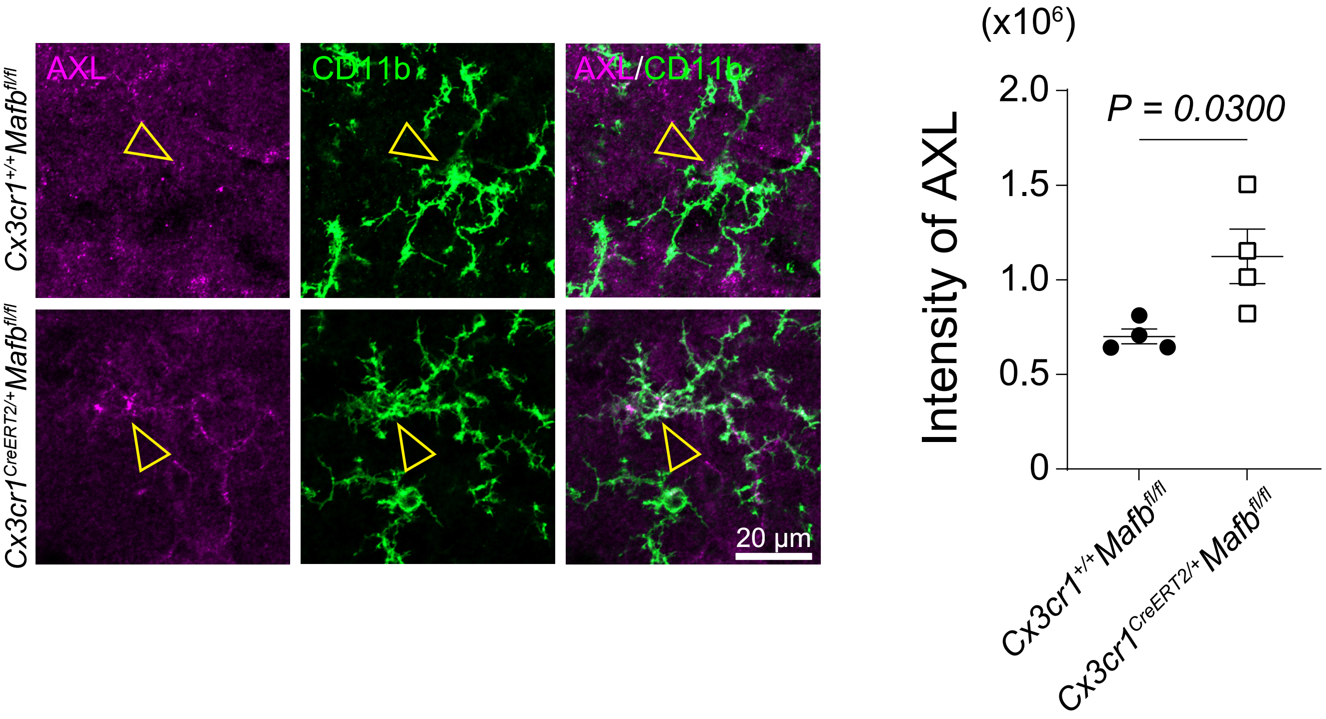

Supplementary Fig. 4: AXL is increased in microglia of *Cx3cr1*$^{CreERT2/+}$*Mafb*$^{fl/fl}$ mice. Representative immunofluorescence images and quantification revealing increased intensiry of AXL in microglia (blank yellow arrowhead) from the cortex of *Cx3cr1*$^{CreERT2/+}$*Mafb*$^{fl/fl}$ compared to *Cx3cr1*$^{+/+}$*Mafb*$^{fl/fl}$.Each symbol represents individual mice (n = 4). Means ± s.e.m. unpaired two-tailed *t*-test.
