# Supplementary material for: IRF8 and MAFB drive distinct transcriptional machineries in different resident macrophages of the central nervous system
Source: Commun Biol. 2024 Jul 24;7:896. doi: 10.1038/s42003-024-06607-6 (PMC11266354; doi:10.1038/s42003-024-06607-6)
Supplement: Supplementary file 2 — Description of additional supplementary files [file 42003_2024_6607_MOESM2_ESM.docx]

Description of Additional Supplementary Files

**File name:** Supplementary Data 1

**Description:** The numerical source data for graphs in the paper.

**File name:** Supplementary Table 1

**Description:** Differentially expressed genes (DEGs) in *Irf8*-deficient microglia and CAMs as shown in Figure 4 and in *Mafb*-deficient microglia and CAMs as shown in Figure 5
