# Supplementary material for: IRF8 and MAFB drive distinct transcriptional machineries in different resident macrophages of the central nervous system
Source: Commun Biol. 2024 Jul 24;7:896. doi: 10.1038/s42003-024-06607-6 (PMC11266354; doi:10.1038/s42003-024-06607-6)
Supplement: Supplementary file 5 — reporting-summary [file 42003_2024_6607_MOESM5_ESM.pdf]

nature portfolio

Corresponding author(s): Takahiro Masuda

Last updated by author(s): Jun 18, 2024

Reporting Summary

Nature Portfolio wishes to improve the reproducibility of the work that we publish. This form provides structure for consistency and transparency in reporting. For further information on Nature Portfolio policies, see our Editorial Policies and the Editorial Policy Checklist.

Statistics

For all statistical analyses, confirm that the following items are present in the figure legend, table legend, main text, or Methods section.

n/a	Confirmed	
☐	☒	The exact sample size (*n*) for each experimental group/condition, given as a discrete number and unit of measurement
☐	☒	A statement on whether measurements were taken from distinct samples or whether the same sample was measured repeatedly
☐	☒	The statistical test(s) used AND whether they are one- or two-sided<br>*Only common tests should be described solely by name; describe more complex techniques in the Methods section.*
☒	☐	A description of all covariates tested
☒	☐	A description of any assumptions or corrections, such as tests of normality and adjustment for multiple comparisons
☐	☒	A full description of the statistical parameters including central tendency (e.g. means) or other basic estimates (e.g. regression coefficient) AND variation (e.g. standard deviation) or associated estimates of uncertainty (e.g. confidence intervals)
☐	☒	For null hypothesis testing, the test statistic (e.g. *F*, *t*, *r*) with confidence intervals, effect sizes, degrees of freedom and *P* value noted<br>*Give P values as exact values whenever suitable.*
☒	☐	For Bayesian analysis, information on the choice of priors and Markov chain Monte Carlo settings
☒	☐	For hierarchical and complex designs, identification of the appropriate level for tests and full reporting of outcomes
☒	☐	Estimates of effect sizes (e.g. Cohen's *d*, Pearson's *r*), indicating how they were calculated

Our web collection on statistics for biologists contains articles on many of the points above.

Software and code

Policy information about availability of computer code

Data collection	Imaging: Zen software (v3.7) was used for confocal imaging with ZEISS LSM 900.<br>IMARIS software was used for morphological analysis.<br>Flow cytometry: CytExpert software was used for flow cytometry analysis with CytoFlex SRT (Beckmam Coulter).
Data analysis	GraphPad Prism v9.5.1 was used for statistical analysis.

For manuscripts utilizing custom algorithms or software that are central to the research but not yet described in published literature, software must be made available to editors and reviewers. We strongly encourage code deposition in a community repository (e.g. GitHub). See the Nature Portfolio guidelines for submitting code & software for further information.

Data

Policy information about availability of data

All manuscripts must include a data availability statement. This statement should provide the following information, where applicable:

- Accession codes, unique identifiers, or web links for publicly available datasets
- A description of any restrictions on data availability
- For clinical datasets or third party data, please ensure that the statement adheres to our policy

The bulk RNA-sequencing data related to Fig. 1 are available in the previous paper9. The other raw data for mouse bulk RNA-sequencing have been deposited in the

Gene Expression Omnibus, and are available at the following accession number: GSE269745. All other data that support the findings of this study are available from the corresponding authors upon reasonable request.

Human research participants

Policy information about studies involving human research participants and Sex and Gender in Research.

Reporting on sex and gender	The study did not involve human participants.
Population characteristics	The study did not involve human participants.
Recruitment	The study did not involve human participants.
Ethics oversight	The study did not involve human participants.

Note that full information on the approval of the study protocol must also be provided in the manuscript.

Field-specific reporting

Please select the one below that is the best fit for your research. If you are not sure, read the appropriate sections before making your selection.

☒ Life sciences ☐ Behavioural & social sciences ☐ Ecological, evolutionary & environmental sciences

For a reference copy of the document with all sections, see nature.com/documents/nr-reporting-summary-flat.pdf

Life sciences study design

All studies must disclose on these points even when the disclosure is negative.

Sample size	No statistical methods were used to predetermine sample sizes. We ensured they were similar to those generally employed in the field for similar methods.
Data exclusions	No data were excluded from the study.
Replication	To be sure of the reproducibility of the experimental findings, we have usually used at least 3 individual mice from at least two independent experiments for each sample group. All replications were successful.
Randomization	Wherever applicable, mice used were randomly allocated into each experimental group. In most experiments, animals were grouped based on genotype or age.
Blinding	During quantification of histological data, researchers were blind to the genotype of the animals whenever different genotypes were compared directory. This was due to the fact that imaging and quantification was done by the same researcher and differences in size. Analysis of sequencing data, or protein expression/fluorescence intensities in flow cytometry was performed in an unbiased way based on software algorithms selection based on appropriate controls.

Reporting for specific materials, systems and methods

We require information from authors about some types of materials, experimental systems and methods used in many studies. Here, indicate whether each material, system or method listed is relevant to your study. If you are not sure if a list item applies to your research, read the appropriate section before selecting a response.

Materials & experimental systems

n/a	Involved in the study	
☐	☒	Antibodies
☒	☐	Eukaryotic cell lines
☒	☐	Palaeontology and archaeology
☐	☒	Animals and other organisms
☒	☐	Clinical data
☒	☐	Dual use research of concern

Methods

n/a	Involved in the study	
☒	☐	ChIP-seq
☐	☒	Flow cytometry
☒	☐	MRI-based neuroimaging

Antibodies

Antibodies used	Primary antibodies flow cytometry:<br>CD11b [M1/70] 1:200 (BV786, BD Bioscience, https://www.bdbiosciences.com/ja-jp/products/reagents/flow-cytometry-reagents/research-reagents/single-color-antibodies-ruo/bv786-rat-anti-cd11b.740861)<br><br>CD45 [30-F11] 1:200 (APC-Cy7, BioLegend, 103116, https://www.biolegend.com/ja-jp/products/apc-cyanine7-anti-mouse-cd45-antibody-2530?GroupID=BLG1932)<br><br>Ly6C [AL-21] 1:200 (PerCP-Cy5.5, BD Biosciences, 560525, https://www.bdbiosciences.com/en-us/products/reagents/flow-cytometry-reagents/research-reagents/single-color-antibodies-ruo/percp-cy-5-5-rat-anti-mouse-ly-6c.560525)<br><br>Ly6G [1A8] 1:200 (PE-Cy7, BD Biosciences, 127618, https://www.bdbiosciences.com/en-us/products/reagents/flow-cytometry-reagents/research-reagents/single-color-antibodies-ruo/pe-cy-7-rat-anti-mouse-ly-6g.560601)<br><br>CD206 [C068C2] 1:100 (APC, Biolegend, 141708, https://www.biolegend.com/ja-jp/products/apc-anti-mouse-cd206-mmr-antibody-7425?GroupID=BLG9506)<br><br>Primary antibodies for histological analysis:<br>Ibal [018-28523] 1:1000 (Wako, 012-28521, https://labchem-wako.fujifilm.com/us/product/detail/W01W0101-2852.html)<br><br>Ibal [Gp311H9] 1:1000 (Synaptic Systems, 234 308, https://www.sysy.com/product/234308)<br><br>CD11b[5C6] 1:500 (Bio-Rad, MCA711G, https://www.bio-rad-antibodies.com/monoclonal/mouse-cd11b-antibody-5c6-mca711.html)<br><br>CD206 [MR5D3] 1:500 (Bio-Rad, MCA2235, https://www.bio-rad-antibodies.com/monoclonal/mouse-cd206-antibody-mr5d3-mca2235.html?f=purified)<br><br>Collagen IV [polyclonal] 1:200 (Millipore, AB769, https://www.merckmillipore.com/DE/de/product/Anti-Collagen-Type-IV-Antibody,MM_NF-AB769)<br><br>AXL [polyclonal] 1:200 (R&D systems, AF854, https://www.rndsystems.com/products/mouse-axl-antibody_af854)<br><br>Secondary antibodies for immunofluorescence were ordered from ThermoFisher Scientific or Jackson Laboratory and added as follows:<br>Alexa Flour® 405 1:1000, Alexa Flour® 488 1:1000, Cy3 1:1000 and Alexa Fluor® 647 1:1000
Validation	All primary antibodies used for flow cytometry, immunofluorescence, immunohistochemistry have been validated for this application in the respective species by the supplier and have been used in previous studies. For validation methods and references please follow the provided links to the product websites. Fluorescence minus one stains or isotype controls were used to define cell popualation-gates for FACS sorting and to define gates for positive/negative selection for all flow cytometry-based quantifications.<br>Further, positive controls were included for every experiment to ensure that the ABs worked in principle.

Animals and other research organisms

Policy information about studies involving animals; ARRIVE guidelines recommended for reporting animal research, and Sex and Gender in Research

Laboratory animals	Transgenic lines including Cx3cr1CreERT2 (Jackson Lab, B6.129P2(Cg)-Cx3cr1tm2.1(cre/ERT2)Litt/WganJ, #021160) and Irf8flox (Jackson Lab, B6.(Cg)-Irf8tm1.1Hm/J, #014175) mice were used in this study. Mafbflox mice28 were kindly provided by Prof. Lisa Goodrich (Department of Neurobiology, Harvard Medical School).
Wild animals	No wild animals were used in this study.
Reporting on sex	Mixed sex mice were used for the experiments and we did not see any sex-biased phenotypes.
Field-collected samples	No field-collected samples were used in this study.
Ethics oversight	An ethics statement is provided in the methods section. The Institutional Animal Care and Use committee review panels at Kyushu University, Japan, have approved all animal experiments

Note that full information on the approval of the study protocol must also be provided in the manuscript.

Flow Cytometry

Plots

Confirm that:

☒ The axis labels state the marker and fluorochrome used (e.g. CD4-FITC).

☒ The axis scales are clearly visible. Include numbers along axes only for bottom left plot of group (a 'group' is an analysis of identical markers).

☒ All plots are contour plots with outliers or pseudocolor plots.

☒ A numerical value for number of cells or percentage (with statistics) is provided.

Methodology

Sample preparation	After taking out from adult mice, brain was homogenized with syringes in HBSS containing 15 mM HEPES buffer and 0.54 % glucose. After spinning down, the pellet at the bottom of the tube was then washed once with FACS Buffer (PBS containing 2 % FBS and 10mM EDTA) before staining. The single cell suspension was used for the staining procedure as described. FC receptors were blocked with Fc Block (2.4G2, BD Biosciences) for 10 min at 4°C before incubation with the primary antibodies. Cells were stained with antibodies directed for 40 min at 4°C.
Instrument	Cells were sorted using a CytoFlex SRT (Beckman Coulter)
Software	Data were acqired with CytExpert software (Beckman Coulter). Post aquisition analysis was perfomed using FlowJo software, version 10.9.0.
Cell population abundance	the cell population abundances are provided in the plots depicting the representative gating strategies of each experiment.
Gating strategy	In all experiments, small debris was removed with the preliminary FSC/SSC gate. Single cells were obtained by doublet exclusion. Further, gating strategies for the respective experiments are provided in the figures.

☒ Tick this box to confirm that a figure exemplifying the gating strategy is provided in the Supplementary Information.
